# Evolution of Polyphenols during Syrah Grapes Maceration: Time versus Temperature Effect

**DOI:** 10.3390/molecules24152845

**Published:** 2019-08-05

**Authors:** Chantal Ghanem, Patricia Taillandier, Ziad Rizk, Nancy Nehme, Jean Pierre Souchard, Youssef El Rayess

**Affiliations:** 1 Department of Agriculture and Food Engineering, School of Engineering, Holy Spirit University of Kaslik, Jounieh, Lebanon; 2 Lebanese Agricultural Research Institute, Fanar Station, P.O. Box 90-1965, Jdeidet El-Metn, Fanar, Lebanon; 3 Université de Toulouse, INPT, UPS, CNRS, Laboratoire de Génie Chimique, 4 Allée Emile Monso, F-31432 Toulouse, France; 4 Faculty of Agricultural Engineering and Veterinary Medicine, Lebanese University, Dekwaneh, Lebanon

**Keywords:** phenolic compounds, maceration, Syrah, tannins, anthocyanins

## Abstract

The effect of maceration time and temperature on the phenolic compounds of Syrah grape musts was studied. Pre-fermentation cold (10 °C) and heat maceration (60, 70 and 80 °C) were applied and compared to traditional maceration (control, 25 °C). The macerations were monitored and the kinetic profile of the maceration was studied by taking samples at 0, 2, 4, 8, 24 and 48 h. The results showed that heat treatment had the most significant effect on the extraction of total polyphenol. A significant loss of anthocyanin content was observed when the maceration was extended beyond eight hours at high temperatures, while longer maceration times seemed to favor the extraction of tannins. A principal component analysis showed that independently of the vinification technique, and for the same grape varieties, different winegrowing regions and harvest years affected the phenolic composition of the grape skin.

## 1. Introduction

The transformation of grape juice into wine is a complex process. The quality of wine obtained depends on such diverse factors as raw material, oenological techniques employed, yeast strain, and ageing. The quality of red wines is largely determined by phenolic compounds, especially anthocyanins and tannins. These compounds constitute a decisive factor in red wine quality and contribute to wine organoleptic characteristics such as color, taste, astringency and bitterness. They also confer to the wine the capacity of aging.

Grape phenolics are mainly localized in the skins and seeds. During winemaking, phenolic compounds and other compounds contained in the grape are transferred to the wine by diffusion due to the establishment of contact between the juice and the solid part of grapes. The diffusion period in winemaking is called maceration. Several factors such as grape variety and maturity; the temperature of must or wine; the duration of juice, grape skin, and seed contact; the concentration of alcohol and sulfur dioxide; and the use of enzymes affect the maceration step. 

In order to achieve organoleptic properties beyond those offered by conventional maceration during fermentation, extended contact with skins may occur before (pre-fermentation extended maceration) or after fermentation (post-fermentation extended maceration). Depending on the temperature levels, the pre-fermentation extended maceration could be divided into two categories: (i) Cold maceration or cold soak for low levels of temperature and (ii) heating maceration for high temperatures. The target of macerations is to improve some important quality characteristics of wines such as color and aroma [1,2,3]. Temperature, skin contact time, wine growing regions and grape varieties are important factors to be considered in the results of the pre-fermentative macerations [4,5]. 

However, information about the evolution of phenolic compounds during the pre-fermentation heating maceration of red grapes varieties is scarce in the literature. Therefore, there is not one single procedure to realize the pre-fermentation heating maceration. For that reason, the purpose of this work was to determine the effect of maceration time and temperature on the polyphenol profile of the Syrah musts using pre-fermentation cold and heat maceration compared to the traditional winemaking scheme (control). The determination of the best couple time/temperature of Syrah grapes maceration was reached by means of statistical multivariate analyses (PCA) giving more information for the correct planning and management of winemaking operations.

## 2. Results

### 2.1. Spectrophotometric Determination of Polyphenols

#### 2.1.1. Total Anthocyanins and Tannins Determination

**Figure 1:**
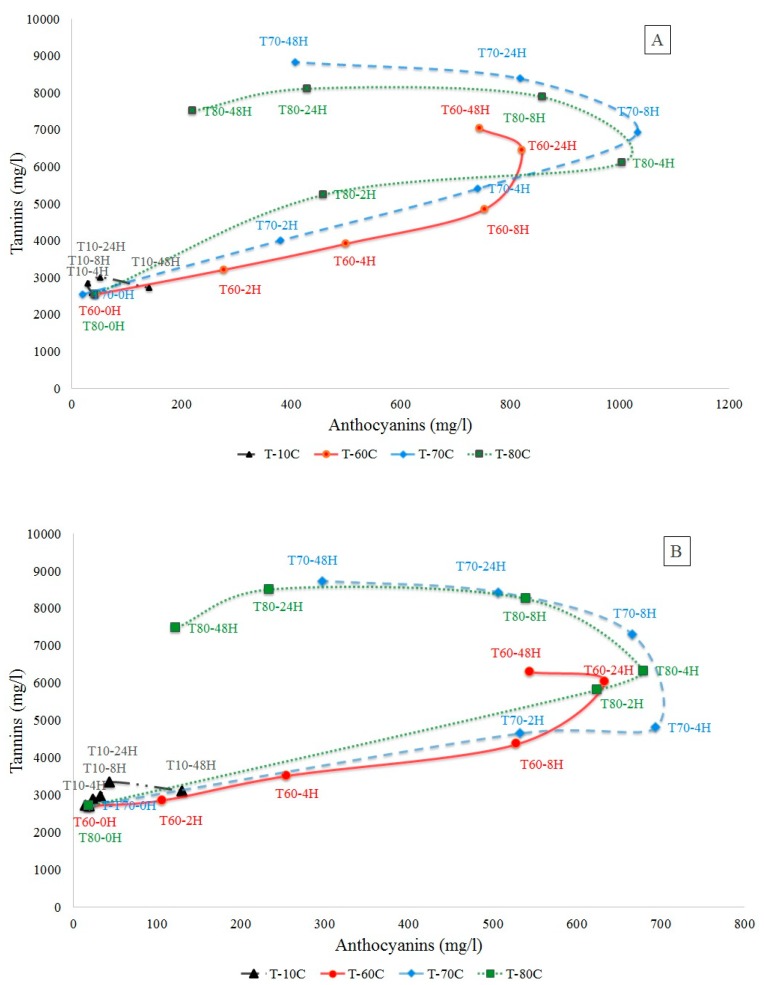

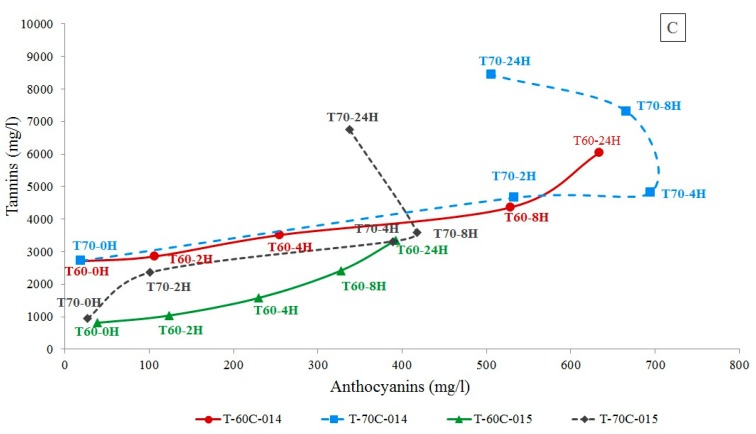
Kinetics of anthocyanins and tannins extraction during the maceration of Syrah grapes from two distinct regions and the two consecutive years (2014 and 2015) in terms of time and temperature (**A**) Chateau Florentine, (**B**) Clos St Thomas 2014, (**C**) Clos St Thomas 2015, T-10C, T-60C, T-70C, T-80C: Maceration temperatures, respectively, at 10, 60, 70 and 80 °C. Example: T-60-4H: Maceration temperature at 60 °C for 4 h.

Figure 1 shows, respectively, the evolution of total tannins versus the total anthocyanins during the maceration of Syrah Florentine (Sy-F) and Syrah Saint Thomas (Sy-ST) (Figure 1A,B) of the 2014 harvest year at different temperatures (10, 60, 70 and 80 °C) for 48 h and the maceration of the 2015 grape harvest year of the Syrah Saint Thomas musts at 60 and 70 °C for 24 h (Figure 1C). The results showed that temperature affects the amounts of tannins and anthocyanins released from skins and seeds to the must. By macerating at 10 °C, the anthocyanin and tannin concentrations increased slightly during the 48 h to reach a maximum of total anthocyanins of 129.79 and 140.29 mg/L, respectively, for the 2014 Sy-ST and Sy-F grape musts. The maximum concentrations of tannins were ([tanins]_Sy-ST_ = 3112.13 mg/L; [tanins]_Sy-F_ = 2719.09 mg/L). Contrariwise, temperature of 60 °C showed a gradual increase in the concentrations of total tannins and anthocyanins (compared to 10 °C) for both grape varieties from the two different regions and years, and the tanins concentration reached its maximum after 48 h ([tanins]_Sy-ST_ = 6301.58 mg/L, ([tanins]_Sy-F_ = 7029.70 mg/L) and for anthocyanins after 24 h ([anthocyanins]_Sy-ST-014_ = 633.79 mg/L, [anthocyanins]_Sy-F_ = 822.79 mg/L, [anthocyanins]_Sy-ST-015_ = 413.10 mg/L). A slight decrease was observed when the heating lasted up to 48 h—the concentrations reached were, respectively, 544.25 and 744.92 mg/L for the 2014 Sy-ST and Sy-F. For temperatures of 70 and 80 °C, a more rapid increase in anthocyanin and tannin concentrations was observed compared to 10 and 60 °C. Similarly, the maximums reached were greater. As for total anthocyanins for the Sy-ST grape must, concentrations reached 694.46 and 680.46 mg/L at 70 and 80 °C, respectively, after 4 h for the 2014 harvest year and 417.37 mg/L at 70 °C after 8 h for the 2015 harvest year. For Sy-F, the maximum concentration was 1033.08 mg/L at 70 °C after 8 h and 1005.60 mg/L at 80 °C after 4 h. Beyond these maximums, a significant decrease in total anthocyanins was observed for the different grape musts and harvest years. This decrease was much greater for 80 than for 70 °C, and concentrations were, respectively, divided by an average factor of 5.05 and 1.79. Concerning tannins concentrations, at 70 °C and for the different grape musts, the maximum tannin extraction was achieved after 48h with a concentration of 8730.72 and 8833.81 mg/L for the 2014 Sy-ST and Sy-F, respectively. For maceration at 80 °C, the maximum extraction was reached faster after 24 h of maceration with a value of 8498.75 mg/L for Sy-ST-014. A slight decrease of 12.13% and 7.45% was noted, respectively, when maceration was prolonged for 48 h for Sy-ST and Sy-F. After alcoholic fermentation, the Sy-ST control (25 °C) showed an anthocyanin concentration of 220.25 mg/L, which is lower than the maximum obtained at 60, 70 and 80 °C. Besides, the Syrah control showed a lower tannin content than the Syrah must from different regions and harvest years macerated at different temperatures (data not shown). 

#### 2.1.2. Total Anthocyanins and Tannins Determination

Table 1 and Table 2 show the evolution of total polyphenol, total polyphenol index and color intensity during the maceration of the Syrah musts from the two different regions and years at different temperatures (10, 60, 70, 80 °C) compared to the control (classical winemaking at 25 °C). For the different grape musts, a slight increase in color intensity was observed at the temperature of 10 °C for 48 h (CI_Sy-ST-014_ = 0.55; CI_Sy-F_ = 0.65). A gradual increase was observed at 60 °C, the color intensity reaching its maximum after 24 h with a value of 1.53; 1.81 and 1.08 for the Sy-ST-014, Sy-F and Sy-ST-015, respectively. However, a high increase in color intensity was observed at 70 °C. This maximum was reached after 24 h for Sy-ST-014 (CI_Sy-ST-014_ = 1.60) and 8 h for Sy-F and Sy-ST-015 (CI_Sy-F_ = 2.61; CI_Sy-ST-015_ = 1.51). A significant increase in the color intensity up to two was observed after 48 h for Sy-ST-015 at 80 °C. Therefore, color intensity showed a similar tendency as anthocyanins, except for the temperature of 80 °C, for which the lower values of anthocyanins were associated with the higher values of color intensity (CI). 

The total polyphenols were characterized qualitatively by the total polyphenols index (TPI) and quantitatively by the analysis of the total polyphenol (TP) by the Folin–Ciocalteu method. The results showed an increase in TPI with temperature and over time (Table 1 and Table 2). A low maceration temperature (10 °C) did not allow for any evolution of TPI over time. After 48 hours of maceration, the TPI values were 22.30; 62.30; 85.20 and 89.20 at 10, 60, 70 and 80 °C, respectively, for the Sy-ST-014 grape must; 18.07; 74.07; 88.20 and 86.80 at 10, 60, 70 and 80 °C, respectively, for the Sy-F-014 grape must; and 42 and 60.47 at 60 and 70 °C, respectively, for the Sy-ST-014 grape must after 24 h. 

Concerning the total polyphenols, a low presence of polyphenols was observed at 10 °C due to an almost non-existent extraction ([TP]_Sy-ST-014_ = 683.33 mg/L gallic acid equivalent (GAE); [TP]_Sy-F-014_ = 606.67 mg/L (GAE), after 48 h). At 60 °C, an improved extraction of polyphenols was observed compared to that carried out at 10 °C. The maximum extraction was reached at 48 h for the 2014 Syrah musts. The maximum concentrations obtained were 2756.70 and 2643.30 mg/L (GAE), respectively, from maceration of the grape musts of Syrah Florentine and Syrah Saint Thomas. At 70 °C, a more rapid increase in total polyphenols was observed with higher maximum concentrations compared to 60 °C. The maximum extraction was 4660 and 4380 mg/L GAE, respectively, for the Sy-F-014 and Sy-St-014 musts after 48 h. After alcoholic fermentation, the Syrah control showed higher values for color intensity, total polyphenol index and total polyphenols than those of the 2014 Syrah musts macerated at 10 °C after 48 h (average values were 2.04; 3.01 and 3.81 times higher, respectively, for CI, TPI and TP) and lower values than that macerated at 60, 70 and 80 °C (average values were 1.24; 1.13 and 1.10 times lower, respectively, for CI, TPI and TP at 60 °C, 1.37; 1.44; and 1.84 times lower, respectively, for CI, TPI and TP at 70 °C and 1.63; 1.46 and 1.30 times lower, respectively, for CI, TPI and TP at 80 °C ). On the other hand, the Syrah control indicated higher values of CI, TPI and TP than the Syrah musts of the 2015 harvest year macerated at 60 °C and lower values than the Syrah must macerated at 70 °C after 24 h.

### 2.2. Chromatographic Determination of Phenolic Compounds

**Figure 2:**
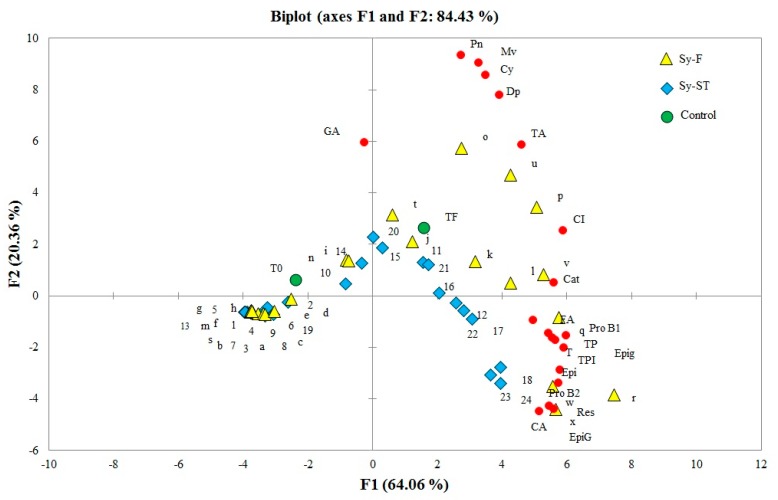
Biplot of the two first principal components obtained from the color and phenolic composition of the Sy-F (Syrah Florentine) and Sy-ST (Syrah Saint Thomas) musts compared to the Syrah Saint Thomas control (Sy-control): TA, total anthocyanin content; CI, color intensity; TPI, total polyphenol index; TP, total polyphenols; T, tannins; Dp, delphinidin-3-O-glucoside; Cy, cyanidin-3-O-glucoside; Pn, peonidin-3-O-glucoside; Mv, malvidin-3-O-glucoside; GA, gallic acid; pro B1, procyanidin B1; EpiG, epigallocatechin; cat, catechin; Pro B2, procyanidin B2; CA, caffeic acid; Epi, epicatechin; Epig, epicatechin gallate; FA, ferulic acid; Res; resveratrol; obtained after maceration at different temperatures (10, 60, 70 and 80 °C) for 48 h (a, Sy-F-0-10 °C; b, Sy-F-2-10 °C, c, Sy-F-4-10 °C; d, Sy-F-8-10 °C; e, Sy-F-24-10 °C; f, Sy-F-48-10 °C; g, Sy-F-0-60 °C; h, Sy-F-2-60 °C; i, Sy-F-4-60 °C; j, Sy-F-8-60 °C; k, Sy-F-24-60 °C; l, Sy-F-48-60 °C; m, Sy-F-0-70 °C; n, Sy-F-2-70 °C; o, Sy-F-4-70 °C; p, Sy-F-8-70 °C; q, Sy-F-24-70 °C; r, Sy-F-48-70 °C; s, Sy-F-0-80 °C; t, Sy-F-2-80 °C; u, Sy-F-4-80 °C; v, Sy-F-8-80 °C; w, Sy-F-24-80 °C; x, Sy-F-48-80 °C; 1, Sy-ST-0-10 °C; 2, Sy-ST-2-10 °C; 3, Sy-ST-4-10 °C; 4, Sy-ST-8-10 °C; 5, Sy-ST-24-10 °C; 6, Sy-ST-48-10 °C; 7, Sy-ST-0-60 °C; 8, Sy-ST-2-60 °C; 9, Sy-ST-4-60 °C; 10, Sy-ST-8-60 °C; 11, Sy-ST-24-60 °C; 12, Sy-ST-48-60 °C; 13, Sy-ST-0-70 °C; 14, Sy-ST-2-70 °C; 15, Sy-ST-4-70 °C; 16, Sy-ST-8-70 °C; 17, Sy-ST-24-70 °C; 18, Sy-ST-48-70 °C; 19, Sy-ST-0-80 °C; 20, Sy-ST-2-80 °C; 21, Sy-ST-4-80 °C; 22, Sy-ST-8-80 °C; 23, Sy-ST-24-80 °C; and 24, Sy-ST-48-80 °C. To, Syrah control at the beginning of maceration; TF, Syrah control at the end of alcoholic fermentation.

**Figure 3:**
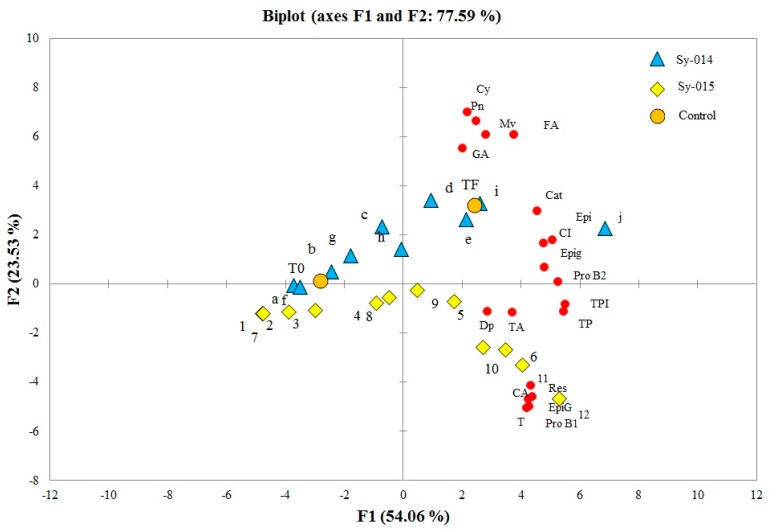
Biplot of the two first principal components obtained from the color and phenolic composition of the 2014 and 2015 Syrah grape harvest years: TA, total anthocyanin content; CI, color intensity; TPI, total polyphenol index; TP, total polyphenols; T, Tannins; Dp, delphinidin-3-O-glucoside; Cy, cyanidin-3-O-glucoside; Pn, peonidin-3-O-glucoside; Mv, malvidin-3-O-glucoside; GA, gallic acid; pro B1, procyanidin B1; EpiG, epigallocatechin; cat, catechin; Pro B2, procyanidin B2; CA, caffeic acid; Epi, epicatechin; Epig, epicatechin gallate; FA, ferulic acid; Res; resveratrol; obtained after maceration at different temperatures for 48 and 24 h, respectively, for 2014 and 2015 vintage (1, Sy-0-60 °C-014; 2, Sy-2-60 °C-014; 3, Sy-4-60 °C-014; 4, Sy-8-60 °C-014; 5, Sy-24-60 °C-014; 6, Sy-48-60 °C-014 7, Sy-0-70 °C-014; 8, Sy-2-70 °C-014; 9, Sy-4-70 °C-014; 10, Sy-8-70 °C-014; 11, Sy-24-70 °C-014; 12, Sy-48-70 °C-014; a, Sy-0-60 °C-015; b, Sy-2-60 °C-015; c, Sy-4-60 °C-015; d, Sy-8-60 °C-015; e, Sy-24-60 °C-015; f, Sy-0-70 °C-015; g, Sy-2-70 °C-015; h, Sy-4-70 °C-015; i, Sy-8-70 °C-015; and j, Sy-24-70 °C-015. T0, Syrah control at the beginning of maceration; TF, Syrah control at the end of fermentation.

In order to illustrate the evolution of flavan-3-ols and non-flavonoids compounds from grapes for extended maceration times and temperatures, Figure 2 and Figure 3, respectively, represent the evolution of the Syrah musts from the two different regions and the two consecutive years over maceration time at different temperatures compared to the Syrah control at the end of alcoholic fermentation. Figure 2 shows the PCA biplot for the first two principal component analyses obtained from the color and phenolic composition of the Sy-F and Sy-ST musts, which explain 80.95% of the total variance. The first component is positively represented by the variables TA (total anthocyanin content), CI, TPI, TP, T (tannins), Pro B1 (procyanidin B1), EpiG (epigallocatechin), Cat (catechin), Pro B2 (procyanidin B2), CA (caffeic acid), Epi (epicatechin), Epig (epicatechin gallate), FA (ferulic acid), and Res (resveratrol). The second component was positively represented by Dp (delphinidin-3-O-glucoside), Cy (cyanidin-3-O-glucoside), Pn (peonidin-3-O-glucoside), Mv (malvidin-3-O-glucoside), and GA (gallic acid), while Figure 3 showed the PCA biplot for the first two principal component analyses obtained from the color and phenolic composition of Sy-ST from two consecutive years, which explain 77.59% of the total variance. The first component is positively represented by the variables TA, CI, TPI, TP, T, Dp, Pro B1, EpiG, Cat, ProB2, C.A, Epi, Epig and Res. The second component is positively represented by FA, Cy, Pn, Mv and GA. The projection of the Syrah Saint Thomas and Syrah Florentine must samples (Figure 2) over maceration time (0, 2, 4, 8, 24 and 48 h) at different temperatures (10, 60, 70, and 80 °C) showed a similar evolution of the two musts over time with a significantly higher concentration of flavonoid and non-flavonoid compounds from the grape musts collected from the Chouf region vineyard (data not shown). Malvidin-3-O-glucoside (Mv) remained the most abundant compound found at different maceration temperatures with a maximum concentration of [Mv] Sy-ST-48 h = 2.78 mg/L, [Mv] Sy-F-48 h = 7.65 mg/L at 10 °C; [Mv] Sy-ST-24 h = 85.39 mg/L, [Mv] Sy-F-24 h = 77.92 mg/L at 60 °C; [Mv] Sy-ST-4 h = 84.77 mg/L, [Mv] Sy-F-6 h = 153.89 mg/L at 70 °C and [Mv] Sy-ST-8 h = 81.74 mg/L, [Mv] Sy-F-4 h = 158.70 mg/L at 80 °C. Following these peaks, a marked decrease was observed over time for the Syrah musts from the two different regions. As for momomeric and dimeric tannins, the extraction of catechin, epicatechin, epicatechin gallate, epigallocatechin, and procyanidin B1 and B2 was favored by high temperatures compared to the low temperature (10 °C) and even when treatment was prolonged over time for the different grape musts. The concentration of these monomers and dimers gradually increased with increasing temperature to maximum values of 45.48 mg/L for Sy-ST-70 °C after 48 h, 158.30 mg/L for Sy-F-80 °C after 48 h, 54.60 mg/L for Sy-F-80 °C after 24 h, 77.59 mg/L for Sy-ST-70 °C after 24 h, 323.23 mg/L for Sy-ST-70 °C after 48 h and 262.62 mg/L for Sy-F-70 °C after 48 h, respectively, for catechin, epicatechin, epicatechin gallate, epigallocatechin, and procyanidin B1 and B2. In addition, concerning hydroxybenzoic acids, the extraction of gallic acid was favored by high temperatures around 4 h for Sy-F and Sy-ST. At 48 h for 60, 70, and 80 °C, gallic acid was no longer detected by liquid chromatography. The obtained results showed that heat promoted caffeic and ferulic acid extraction compared to the low temperature (10 °C). The maximum extraction was obtained for 48 h at 60, 70 and 80 °C. Syrah Florentine showed the max concentration of ferulic acid (48.40 mg/L) after 48 h at 70 °C and caffeic acid (24.80 mg/L) after 48 h at 80 °C. After all, the extraction of resveratrol increased progressively as temperature increased over the time to reach a concentration 2.37 times higher for Sy-F (50.90 mg/L, 70 °C, 48 h) than for Sy-ST (21.47 mg/L, 80 °C, 48 h). Additionally, all the tannin compounds revealed an increase in concentration with temperature and macerating time, which correlates with the values of total polyphenols obtained by spectrophotometric determinations.

The projection of the 2014 and 2015 grape harvest year of the Syrah Saint Thomas must samples (Figure 3) over maceration time (0, 2, 4, 8, and 24 h) at different temperatures (60, 70 and 25 °C), showed similar evolution over time for the two consecutive years with different concentrations in phenolic compounds. As seen previously (Figure 2), malvidin-3-O-glucoside remained the most represented compound with a maximum concentration of 88.24 mg/L for Sy-2015-70 °C after 4 h. With few exceptions, the 2014 Syrah musts showed significantly higher anthocyanins profiles than those of the 2015 harvest year. With regard to monomeric tannins, dimeric tannins, and phenolic acids, their extraction was favored by higher temperatures (70 °C). In terms of concentration, epicatechin was the most represented monomer of flavan-3-ols. With few exceptions, the 2015 Syrah musts showed significantly higher values of catechin ([cat]_Sy_-_015_ = 56.82 mg/L at 60 °C and 108.72 mg/L at 70 °C), epicatechin ([epi]_Sy_-_015_ = 101.06 mg/L at 60 °C and 135.33 mg/L at 70 °C), procyanidin B2 ([pro B2]_Sy_-_015_ = 101.89 mg/L at 60 °C and 134.21 mg/L at 70 °C), gallic acid ([GA]_Sy_-_015_ = 12.16 mg/L at 60 °C and 29.45 mg/L at 70 °C) and ferulic acid ([FA]_Sy_-_015_ = 57.11 mg/L at 60 °C and 80.20 mg/L at 70 °C) than for the 2014. Eventually, regarding stilbenes, the 2014 Syrah musts indicated significantly higher values of resveratrol, which was, on average, a value almost twice as high than for the 2015 musts.

## 3. Discussion

Concerning the total anthocyanins and tannins results, the decrease in anthocyanins could be explained by the thermal degradation of anthocyanins at high temperatures and a shift in the equilibrium towards chalcone and colorless forms [6], the oxidative cleavage of the heterocyclic ring leading to direct anthocyanin degradation [7,8], and the different reactions involving anthocyanins during the extended maceration time [9,10]. In opposition, longer maceration times seemed to favor the extraction of tannins, because the release of these compounds occurred from the grape skins and seeds. In the seeds, flavan-3-ols are located in thin-walled cells between the external hydrophobic cuticle and the inner lignified layers, so the release of these compounds from the seeds requires longer maceration times and high temperatures [11]. Additionally, an increase of color intensity was observed even with the decrease of anthocyanins, which could have been due to the phenomenon of copigmentation. This latter is due to molecular associations between pigments and other organic molecules in solution. These associations cause the pigments to exhibit far greater color than would be expected from their concentration [6].

The increase of phenolic compounds during maceration time can be explained by the fact that the heat destroys the skins cell membranes, releasing the pigments, tannins and different phenolic substances into the must [12].

The results obtained from Florentine Vineyard showed higher concentrations of flavonoid and non-flavonoid compounds than Chateau St Thomas, where several factors such as as light, water deficit, higher temperature differences between daytime and nighttime, training system of the vines, fertilization of soils, irrigation during summer, and canopy management could have played an important role.

Regarding monomeric anthocyanins, cyanidin derivatives showed the lowest concentration, probably because this anthocyanin is the precursor of all others [13].

As seen from our results and the literature, there was a negative relationship between maceration length and anthocyanins monomers concentration in the wines. The influence of temperature on anthocyanins has been studied through the thermal degradation of anthocyanins for blackberry [14], grape pomace [15], and plums [16]. These studies showed that the thermal degradation of anthocyanins followed a first order reaction: C_t_ = C_0_ exp (−kt), where C_t_ is anthocyanin concentration at time t of heating (min), C_0_ is initial concentration of anthocyanins, and K (min^−1^) is the first order kinetic constant. 

The high concentrations of epigallocatechin indicate that the skin tannins were extracted preferentially during the first hours of maceration. while the release of flavan-3-ols from the seeds required longer maceration times or the presence of ethanol [11].

The highest degree of ripening of the 2015 harvest year compared to 2014 was not correlated with the higher concentrations of phenolic compounds. In fact, an unseasonal sandstorm hit the Bekaa valley in eastern Lebanon. These climatic conditions could have induced damage in anthocyanins and tannins, reducing their amounts. Other studies conducted by [17] indicated that sunlight exposure (other climatic conditions), essential for grape berry ripening, could also be responsible for excessive sunburn and qualitative and quantitative vine damages, especially on anthocyanins accumulation of Nebbiolo grapes skins. After all, while tannins were progressively extracted from skins and seeds, the potential of anthocyanins was extracted since the first hours, so the temperature and length of maceration must be adjusted to grape musts according to the defined region and year. Moreover, Figure 2 allowed for the establishment of the best coupled time/temperature for each grape must without the degradation kinetics of anthocyanins and gallic acid over time. This couple was represented by the letter v (Figure 2) corresponding to Sy-F-8-80 °C and the numbers 12 and 16 (Figure 2) corresponding, respectively, to Sy-ST-48-60 °C and Sy-ST-8-70 °C.

## 4. Materials and Methods

### 4.1. Chemicals and Standards

All chemicals used were of analytical reagent grade. All chromatographic solvents (acetonitrile, acetic acid) were high-performance liquid chromatography (HPLC) grade and were purchased from Sigma-Aldrich (Steinheim, Germany). Delphinidin 3-*O*-glucoside, cyanidin 3-*O*-glucoside, peonidin-3-O-glucoside, malvidin 3-O-glucoside, (+)-catechin, (−)-epicatechin, (−)-epicatechin gallate (−)-epigallocatechin, (−)- epigallocatechin gallate, procyanidin B1, procyanidin B2, ferulic acid, caffeic acid and trans-resveratrol were purchased from Extrasynthèse (Lyon, Genay-France). The Folin–Ciocalteu reagent was obtained from Sigma-Aldrich (Steinheim, Germany).

### 4.2. Samples

Red grapes of *Vitis vinifera* var. Syrah (Sy) were supplied by two cellars from distinct regions: Clos St. Thomas (West Bekaa/Lebanon) for two consecutive years—2014 and 2015—and Chateau Florentine (Chouf District/Lebanon) for the 2014 year. Regarding the data obtained from the 2014 maceration campaign, the maceration campaign for the 2015 harvest year was restricted to the Saint Thomas cellar. Table 3 summarizes the soil type and regional climate conditions of each studied region. Grapes were harvested in 2014 and 2015 at technological maturity (Brix = 21.2 and 23.2; titrable acidity = 4.4 and 3.7 g/L as sulfuric acid for 2014 Syrah Saint Thomas and Syrah Florentine, respectively), and (degree Brix = 22.4; titrable acidity = 3.6 g/L as sulfuric acid for 2015 Syrah Saint Thomas).

### 4.3. Strains and Storage Conditions

*S. cerevisiae* Y were used in this work and were kindly provided by Lallemand Inc. (Blagnac, France), kept anonymous according to the supplier’s request. Y strain enhances varietal wine aromas for the Burgundy regions. Yeast stock cultures were kept at 4 °C in YEPD (yeast extract peptone dextrose) agar slants composed of 10 g/L Yeast extract, 20 g/L peptone, 20 g/L D-glucose and 20 g/L agar. The yeast inoculum was previously prepared in two steps. First, a preculture of the yeast strain was obtained by reactivating the stock culture in YEPD broth for 24 h. Second, the preculture was used to inoculate a low sugar concentration synthetic grape juice medium composed of 50 g/L D-glucose, 1 g/L yeast extract, 2 g/L ammonium sulfate, 0.3 g/L citric acid, 5 g/L L-malic acid, 5 g/L L-tartaric acid, 0.4 g/L magnesium sulfate, and 5 g/L potassium dihydrogen phosphate. This step was carried out for 48 h and provided the yeast inoculum.

### 4.4. Maceration and Fermentation Procedures and Sampling 

After reception, the grapes were crushed and destemmed manually, and sodium metabisulphite was added (5 g of NaHSO_3_/100 kg). Two kilogram lots of grapes were drawn into glass Erlenmeyer flasks of 2 L, and the pre-fermentative macerations were conducted at different temperatures (10, 60, 70 and 80 °C) for 48 h for the 2014 harvest year. The macerations were monitored and the kinetic profile of the maceration was studied by taking samples at 0, 2, 4, 8, 24 and 48 h. Based on data collected from the maceration part of the 2014 grape harvest year, temperatures of 10 and 80 °C were abandoned for the 2015 harvest year, and maceration time was restricted to 24 h (samples taken at 0, 2, 4, 8 and 24 h). Classical winemaking process (maceration and fermentation occur together at 25 °C) of 2014 Syrah Saint Thomas were used as controls. Musts issued from the control were separately inoculated by *S. cerevisiae* Y yeast strain at an initial concentration of 3 × 10^6^ cells/mL (Thoma cell counting chamber). The alcoholic fermentation was followed until the total cessation of sugar consumption (<2 g/L, DNS colorimetric method) [18], and finished after 10 days. Control samples were collected at the end of the alcoholic fermentation. A volume of 50 mL of each sample was collected and directly centrifuged for 5 min at 2795 g. The samples were stored at 0 °C until analyses. All macerations and fermentations were run in triplicate. 

### 4.5. Spectrophotometric Determinations of Polyphenols

The color intensity (CI) defined as the sum of absorbencies at 420 and 520 [19].

The total anthocyanins were calculated by measurement of the absorbance at 520 nm after bleaching with sodium bisulfite solution (15%). Total anthocyanin concentration was expressed in mg/L, as described by Ribéreau-Gayon and Stonestreet (1965) [20].

The total polyphenols index (TPI) was determined following the method described by Ribéreau-Gayon et al. (1998) [21]. Wines were diluted with water (1:100), and the absorbance was measured directly at 280 nm.

Total phenolics were determined using the Folin–Ciocalteu colorimetric method [22], and the results were expressed as gallic acid equivalent (mg GAE/L).

Total tannins were determined by absorbance measurement at 550 nm after the acid hydrolysis of the samples and a blank. The total tannins concentration was expressed in mg/L, as described by Ribéreau-Gayon and Stonestreet (1965) [23]. 

### 4.6. HPLC Analysis of Phenolic Compounds

The HPLC analyses were performed using a Shimadzu chromatographic system equipped with a quaternary pump (LC-20AD), a UV-Vis diode-array detector (SPD-M20A), an automatic injector (SIL-20A), and Shimadzu LC solution software. The method was previously described by Ghanem et al. (2017) [24]. Chromatograms were recorded at 520, 280 and 320 nm for anthocyanins, flavan-3-ols and phenolics acids, respectively. Calibration curves were obtained for all phenols standards, and the concentrations were expressed as mg/L.

### 4.7. Statistical Data Treatment

All experiments were carried out in triplicate. An analysis of variance (ANOVA) and Tukey’s honestly significant difference (HSD) test were used for mean separation, with a significant level of 95% (*p* ˂ 0.05). These statistical analyses, together with PCA, were conducted using Xlstat software (2014).

## 5. Conclusions

The results presented in this study highlight that the phenolic composition of musts is greatly affected by the maceration step. The pre-fermentation heat treatment of grapes is more efficient for the extraction of polyphenols than the cold maceration. An analysis of must samples revealed a systematic increase in the concentration of tannins with temperature and over time. Temperature favored anthocyanin extraction, but a degradation of these compounds was observed at high temperatures when the maceration was extended beyond eight hours. Results from PCA showed that temperature and length of maceration are parameters that must be adjusted to grape musts depending on defined region and year. At the end, due to some particular weather conditions, the 2014 Syrah musts showed higher total polyphenol content than 2015.

## Figures and Tables

**Table 1:** Total polyphenol, total polyphenol index and color intensity of the 2014 Syrah Saint Thomas and Syrah Florentine musts and the 2014 grape harvest year of the Syrah Saint Thomas control in terms of time and temperature.

Sy Maceration Time (Hours)														
			0		2		4		8		24		48	
		Control 25 °C	F	ST	F	ST	F	ST	F	ST	F	ST	F	ST
10 °C	CI	1.22 ± 0.01	0.42 ± 0.01 ^a^	0.34 ± 0.01 ^b^	0.43 ± 0.10 ^a^	0.42 ± 0.21 ^a^	0.44 ± 0.10 ^a^	0.46 ± 0.01 ^a^	0.49 ± 0.00 ^a^	0.49 ± 0.00 ^a^	0.59 ± 0.00 ^a^	0.52 ± 0.00 ^b^	0.65 ± 0.00 ^a^	0.55 ± 0.00 ^b^
	TPI	60.12 ± 2.57	16.20 ± 1.08 ^a^	16.27 ± 0.50 ^a^	16.22 ± 0.65 ^b^	19.10 ± 0.70 ^a^	16.27 ± 0.14 ^b^	19.77 ± 0.78 ^a^	16.30 ± 0.15 ^b^	19.30 ± 0.96 ^a^	15.27 ±1.62 ^b^	20.93 ± 0.94 ^a^	18.07 ± 1.70 ^b^	22.30 ± 0.87 ^a^
	TP	2452.25 ± 46.19	628.33 ± 1.21 ^a^	440.00 ± 0.50 ^b^	605.00 ± 2.89 ^a^	566.67 ± 0.40 ^b^	583.00 ± 1.73 ^a^	521.67± 0.81 ^b^	555.00 ± 2.89 ^a^	573.30 ± 6.07 ^a^	540.00 ± 3.22 ^b^	663.33 ± 3.09 ^a^	606.67 ± 2.89 ^b^	683.33 ± 0.20 ^a^
60 °C	CI	1.22 ± 0.01	0.42 ± 0.01 ^a^	0.34 ± 0.00 ^b^	0.80 ± 0.01 ^a^	0.611 ± 0.03 ^b^	1.20 ± 0.11 ^a^	0.99 ± 0.02 ^a^	1.67 ± 0.04 ^a^	1.34 ± 0.10 ^b^	1.81 ± 0.16 ^a^	1.53 ± 0.01 ^b^	1.80 ± 0.03 ^a^	1.24 ± 0.09 ^b^
	TPI	60.12 ± 2.57	16.93 ± 0.45 ^a^	16.27 ± 0.25 ^a^	26.80 ± 0.05 ^a^	21.97 ± 0.50 ^b^	38.30 ± 0.10 ^a^	29.97 ± 2.90 ^b^	52.15 ± 0.02 ^a^	35.17 ± 2.80 ^b^	64.53 ± 1.81 ^a^	52.93 ± 1.62 ^b^	74.07 ± 1.55 ^a^	62.30 ± 0.63 ^b^
	TP	2452.25 ± 46.19	628.33 ± 0.14 ^a^	441.67 ± 0.81 ^b^	927.20 ± 3.62 ^a^	680.00 ± 3.41 ^b^	1210.80 ± 0.10 ^a^	873.30 ± 4.52 ^b^	1648.90 ± 4.87 ^a^	1393.33 ± 2.51 ^b^	2490.00 ± 0.05 ^a^	2266.67 ± 5.12 ^a^	2756.70 ± 1.66 ^a^	2643.30 ± 2.58 ^a^
70 °C	CI	1.22 ± 0.01	0.42 ± 0.00 ^a^	0.34 ± 0.00 ^b^	1.19 ± 0.11 ^a^	1.30 ± 0.04 ^a^	1.83 ± 0.02 ^a^	1.39 ± 0.10 ^b^	2.61 ± 0.00 ^a^	1.59 ± 0.04 ^b^	2.44 ± 0.09 ^a^	1.60 ± 0.02 ^b^	2.03 ± 0.01 ^a^	1.30 ± 0.06 ^b^
	TPI	60.12 ± 2.57	16.53 ± 0.40 ^a^	16.70 ± 0.10 ^a^	30.60 ± 0.35 ^b^	37.43 ± 0.80 ^a^	45.20 ± 0.15 ^b^	49.93 ± 4.30 ^a^	62.10 ± 0.46 ^a^	56.00 ± 1.30 ^b^	71.80 ± 1.14 ^a^	73.73 ± 2.47 ^a^	88.20 ± 1.80 ^a^	85.20 ± 1.67 ^a^
	TP	2452.25 ± 46.19	628.30 ± 3.63 ^a^	440.00 ± 1.41 ^b^	1296.60 ± 3.44 ^b^	1526.67 ± 1.92 ^a^	1878.40 ± 4.78 ^b^	2155.00 ± 2.74 ^a^	2656.60 ± 3.44 ^b^	2758.33 ± 1.30 ^a^	3185.00 ± 7.55 ^b^	3585.00 ± 1.97 ^a^	4660.00 ± 0.81 ^a^	4380.00 ± 1.39 ^b^
80 °C	CI	1.22 ± 0.01	0.42 ± 0.01 ^a^	0.34 ± 0.01	1.45 ± 0.62 ^a^	1.47 ± 0.04 ^a^	2.63 ± 0.01 ^a^	1.52 ± 0.05 ^b^	2.40 ± 0.00 ^a^	1.66 ± 0.06 ^b^	2.01 ± 0.17 ^a^	1.93 ± 0.06 ^a^	1.95 ± 0.02 ^b^	2.031 ± 0.02 ^a^
	TPI	60.12 ± 2.57	16.70 ± 0.11 ^a^	16.37 ± 0.28	42.30 ± 0.11 ^b^	45.47 ± 0.32 ^a^	58.70 ± 0.11 ^b^	60.87 ± 1.15 ^a^	72.80 ± 0.40 ^a^	73.17 ± 0.17 ^a^	80.50 ± 0.29 ^b^	85.80 ± 1.15 ^a^	86.80 ± 0.00 ^b^	89.20 ± 0.70 ^a^
	TP	2452.25 ± 46.19	628.30 ± 4.20 ^a^	440.00 ± 0.79	1852.00 ± 1.00 ^b^	1875.00 ± 1.22 ^a^	2732.60 ± 3.86 ^b^	2823.33 ± 0.30 ^a^	3108.70 ± 4.71 ^b^	3301.67 ± 1.05 ^a^	3542.80 ± 1.44 ^b^	3661.67 ± 0.50 ^a^	3329.60 ± 5.54 ^a^	3031.67 ± 3.05 ^b^

Mean (n = 3) ± SD. For each maceration time from the two distinct regions, different letters in the same row indicate significant difference at *p* < 0.05. CI, color intensity; TPI, total phenolic index; TP, total phenolic; ST, Saint Thomas; F, Florentine.

**Table 2:** Total polyphenol, total polyphenol index and color intensity of the Syrah Saint Thomas musts for the two consecutive years (2014 and 2015) and the 2014 grape harvest year of the Syrah Saint Thomas control (25 °C) in terms of time and temperature.

Sy Maceration Time (Hours)												
			0		2		4		8		24	
		Control 25 °C	ST-014	ST-015	ST-014	ST-015	ST-014	ST-015	ST-014	ST-015	ST-014	ST-015
60 °C	CI	1.22 ± 0.01	0.34 ± 0.00 ^a^	0.15 ± 0.03 ^b^	0.611 ± 0.03 ^a^	0.39 ± 0.01 ^b^	0.99 ± 0.02 ^a^	0.79 ± 0.03 ^b^	1.34 ± 0.10 ^a^	1.06 ± 0.06 ^b^	1.53 ± 0.01 ^a^	1.08 ± 0.06 ^b^
	TPI	60.12 ± 2.57	16.27 ± 0.25 ^a^	12.50 ± 0.20 ^b^	21.97 ± 0.50 ^a^	16.30 ± 0.56 ^b^	29.97 ± 2.90 ^a^	22.97 ± 1.25 ^b^	35.17 ± 2.80 ^a^	28.60 ± 0.91 ^b^	52.93 ± 1.62 ^a^	42.00 ± 2.19 ^b^
	TP	2452.25 ± 46.19	441.67 ± 0.81 ^a^	401.67 ± 2.87 ^b^	680.00 ± 3.41 ^a^	621.67 ± 5.77 ^b^	873.30 ± 4.52 ^a^	803.33 ± 79.73 ^b^	1393.33 ± 2.51 ^a^	1310.33 ± 18.92 ^a^	2266.67 ± 5.12 ^a^	2172.67 ± 28.43 ^b^
70 °C	CI	1.22 ± 0.01	0.34 ± 0.00 ^a^	0.16 ± 0.02 ^b^	1.30 ± 0.04 ^a^	0.66 ± 0.02 ^b^	1.39 ± 0.10 ^a^	1.16 ± 0.06 ^b^	1.59 ± 0.04 ^a^	1.51 ± 0.09 ^b^	1.60 ± 0.02 ^a^	1.46 ± 0.09 ^b^
	TPI	60.12 ± 2.57	16.70 ± 0.10 ^a^	12.33 ± 0.21 ^b^	37.43 ± 0.80 ^a^	32.37 ± 0.46 ^b^	49.93 ± 3.30 ^a^	40.40 ± 0.26 ^b^	56.00 ± 1.30 ^a^	45.30 ± 0.62 ^b^	73.73 ± 2.47 ^a^	60.47 ± 1.97 ^b^
	TP	2452.25 ± 46.19	440.00 ± 1.41 ^a^	402.67 ± 12.58 ^a^	1526.67 ± 1.92 ^a^	1475.00 ± 13.23 ^b^	2155.00 ± 2.74 ^a^	2051.67 ± 2.88 ^a^	2758.33 ± 1.30 ^a^	2576.67 ± 5.77 ^a^	3585.00 ± 1.97 ^a^	3468.33 ± 2.88 ^a^

Mean (n = 3) ± SD. For each maceration time from the two consecutive years, different letters in the same row indicate significant difference at *p* < 0.05. CI, color intensity; TPI, total phenolic index; TP, total phenolic; ST-014, Syrah Saint Thomas 2014; ST-015, Syrah Saint Thomas 2015.

**Table 3:** Wine producer, regional climate condition and soil type from the two different wine-growing regions.

Wine-Growing Region	Wine-Producer	Soil Type	Climate Condition (2014 Data)
West Bekaa/Lebanon	Clos St. Thomas	Limestone, pebbly clay, clay-calcareous well drained, poor in humus and organic matter	The vineyards are located on the valley zones at the altitude of 950 m with a cool and semi-arid dry climate and a big difference between day and night time, with an annual rainfall of 650 mm, annual average temperature of 21.1 °C.
Chouf District/Lebanon	Florentine	Clay-calcareous, stony basement	The vineyards are located on the mountain’s hills at the altitude of about 1000 m with a warm, dry sub-humid and temperate climate, with an annual rainfall of 1078 mm and annual average temperature of 15.1 °C.
